# A Single-Pass Approach That Mines Unstructured Robotic Trajectories for Calibrating Surveillance Cameras

**DOI:** 10.3390/s26175473

**Published:** 2026-08-29

**Authors:** Wayne Lam, Yingqi Liu, Chong Di, Hao Tang, Jie Gong, Fred Roberts, Zhigang Zhu

**Affiliations:** 1 Department of Computer Science, The Graduate Center—CUNY, New York, NY 10016, USA; wlam1@gc.cuny.edu; 2 Department of Computer Science, The City College of New York—CUNY, New York, NY 10031, USA; yingqi.liu30@stu-mail.ccny.cuny.edu; 3 Department of Civil and Environmental Engineering, Rutgers University, New Brunswick, NJ 08854, USA; cd780@scarletmail.rutgers.edu (C.D.); jg931@soe.rutgers.edu (J.G.); 4 Department of Computer Information Systems, Borough of Manhattan Community College—CUNY, New York, NY 10007, USA; htang@bmcc.cuny.edu; 5 Department of Mathematics, Rutgers University, New Brunswick, NJ 08854, USA; froberts@rutgers.edu

**Keywords:** smart surveillance, robot navigation, camera calibration, assistive technology

## Abstract

The ubiquity of surveillance networks in public infrastructure presents a significant, yet underutilized, opportunity to assist vulnerable populations, particularly individuals who are blind or have low vision (BLV). However, transforming these passive video feeds into active guidance systems requires accurate camera calibration, a process that is traditionally labor-intensive and unscalable in large facilities. This paper introduces a novel, automated framework that leverages a mobile quadruped robot (Boston Dynamics Spot) as a dynamic calibration agent. We propose a single-pass approach with two planar calibration algorithms, which mines unstructured robotic trajectories to construct distinct geometric features on the ground plane. By synthesizing “virtual rectangles” from the robot’s odometry, our method first recovers camera focal length through vanishing point estimation by constructing virtual rectangles, and then solves for extrinsic 6-DoF pose using two algorithms: our proposed Virtual Rectangle (ViR) algorithm and a standard planar Perspective-n-Point (PnP) algorithm. Experimental validation using real-world data demonstrates the system’s robustness to sensor noise, maintaining focal length errors around 5%, rotational errors around $5^{{}^{\circ}}$ and relative translation errors of approximately 10%, while synthetic simulations indicate focal length errors generally under 10%, rotational errors under $1.5^{{}^{\circ}}$, and translation errors also around 10% despite heavy image and object disturbances. This work eliminates the need for manual calibration targets for dynamic camera calibration, effectively converting static security infrastructure into a metric sensing network capable of supporting high-fidelity social robotics applications.

## 1. Introduction

The proliferation of video surveillance in public facilities, such as museums, transit hubs, and sports arenas, is fundamental to managing large-scale environments. Traditionally, these systems rely on human operators to monitor activity, a process that is inherently limited by fatigue, high cognitive load, and the difficulty of identifying specific needs within dense crowds. While these systems are often designed for general safety, there is a significant opportunity to repurpose this infrastructure to assist vulnerable populations. Specifically, individuals who are blind or have low vision (BLV) face immense challenges navigating complex, high-traffic public settings [1,2]. By leveraging existing surveillance infrastructure, we can provide real-time, high-fidelity guidance to improve their autonomy and safety.

Current research in robotics has begun to move beyond simple anomaly detection toward the identification of social cues and the needs of specific demographics, such as people with disabilities. Recent advances in AI, particularly the integration of quadruped robots with Large Language Models (LLMs), have opened new avenues for social impact. These technologies show great promise in applications ranging from guided museum tours to specialized navigational assistance for the visually impaired. However, several technical hurdles remain.

First, the vast majority of cameras in public facilities remain uncalibrated. In large-scale environments like museums and stadiums, it is a daunting task to manually calibrate dozens of cameras. Second, the use of mobile robots as active agents to calibrate these static cameras while simultaneously providing real-time assistance to BLV individuals [3,4] is a dual-purpose strategy that remains largely underexplored. Finally, there is a critical need for convergence between robotic platforms and surveillance camera network in a large public facility.

To address these challenges, this paper introduces a novel technique that utilizes a quadruped robot (Boston Dynamics Spot) [5] as a mobile calibration agent. By adopting a LiDAR-based (Light Detection and Ranging) simultaneous localization and mapping (SLAM) algorithm carried by a highly maneuverable robotic dog platform, we are able to retrieve accurate 3D pose information as well as a point cloud map of its surrounding environment from the robot in real-time so that a homography can be established between the global map coordinates and the image plane from the target camera. Our approach leverages the planar constraints of the robot’s trajectory to recover focal length—without the need for the robot to walk with 3D trajectories—in a typical situation with planar traversal paths such as a museum, a transit hub or a sports arena, by constructing “virtual rectangles” and utilizing geometric constraints of their vanishing points to estimate focal length, while allowing the flexibility of utilizing any robust planar algorithms, such as our proposed “Virtual Rectangle” (ViR) algorithm, or the standard planar Point-n-Perspective algorithm [6], to estimate the camera’s extrinsic parameters. This allows for a “single-pass” unified algorithmic pipeline that extracts both focal length and extrinsic parameters without requiring a separate dedicated calibration run with static targets, or multiple passes through the data. Following a one-time, offline manual setup (which involves training an object detection model for the specific robot), our system matches the robot’s high-precision 3D global points to its 2D coordinates identified in the surveillance feed as it traverses a public environment. This eliminates the need for further pre-calibration or manual intervention, providing an automatic calibration of subsequent cameras on the fly. This creates infrastructure capable of supporting social applications, such as the real-time guidance of individuals with visual impairments.

The primary contributions of this work are as follows:

1. A Single-Pass Planar Calibration Approach: We propose a method to calibrate static infrastructure cameras by matching 2D image features with 3D global coordinates acquired dynamically by a mobile quadruped robot from a single trajectory.
2. Robotic 3D Data Acquisition: We demonstrate the utility of using a quadruped robot to efficiently gather metric-scaled 3D points in indoor environments.

### 1.1. Related Work: Camera Calibration

Camera calibration is a foundational problem in computer vision, traditionally solved by observing known geometric patterns manually designed as calibration targets. The method proposed by Zhang [7] remains the industry standard, utilizing a planar checkerboard to estimate both intrinsic and extrinsic parameters. However, such methods are non-scalable in large public facilities like museums. The labor required to present calibration targets to hundreds of distributed sensors is prohibitive and the process must be repeated whenever a camera is repositioned. Additionally, pan–tilt–zoom (PTZ) cameras are often employed in such facilities and would require the recalibration of focal length estimation and camera pose after changing orientation or optical zoom.

A common alternative in robotics is the Point-n-Perspective (PnP) estimation, which determines the pose of a camera given a set of 3D global points and their corresponding 2D image projections. While general algorithms such as EPnP [8] or P3P [9] are computationally efficient, they are generally “extrinsic-only” solvers; they assume that the camera’s intrinsic parameters (focal length and principle point/optical center) are already known or have been calibrated a priori. In heterogeneous camera networks where cameras vary in model, resolution, and zoom level, this requirement for pre-known focal length represents a significant barrier to automated calibration.

In recent studies, much work is focused on calibrating heterogeneous traffic camera networks. In [10], external cameras, such as pre-calibrated Google Street View images, are leveraged to reconstruct a 3D space and estimating the camera pose of target traffic cameras embedded in the reconstructed 3D space. Beyond the calibration of traffic cameras, the authors of [11] propose using ceiling-mounted LED grids and calculating the shape of their ground reflection to estimate smartphone camera poses, while ref. [12] calibrates security cameras by utilizing rigid objects in place of calibration patterns.

Acquiring reliable 2D image coordinates is a critical prerequisite for visual–spatial tasks. Traditional approaches typically extract 2D observation points using artificial fiducial markers, such as checkerboards or AprilTags [13], or classical corner detection algorithms. While effective, these methods strictly depend on the deployment of physical markers or environments with rich textures. To eliminate the need for artificial markers, recent pipelines increasingly adopt deep learning-based object detectors to localize specific targets directly from images. In this paper, since the 2D feature localization itself is not our primary contribution but rather a necessary step to acquire observations, we directly employ a state-of-the-art detector, YOLOv8 [14], as an efficient tool to achieve robust and automatic target localization in our pipeline.

### 1.2. Related Work: LiDAR-Based Simultaneous Localization and Mapping (SLAM)

LiDAR-based state estimation has evolved from classical point cloud registration to increasingly direct, feature-free formulations. Early scan-matching methods such as iterative closest point (ICP) [15], point-to-plane ICP [16], and Generalized-ICP (GICP) [17] established the registration primitives underlying most later systems, with GICP’s probabilistic point-to-plane formulation forming the basis of the direct methods central to this work. Building on these primitives, LiDAR odometry and mapping (LOAM) [18] introduced real-time LiDAR odometry by extracting sparse edge and planar features and matching them across scans. LOAM was later refined by LeGO-LOAM [19] and F-LOAM [20] for efficiency and ground-vehicle deployment. LiDAR-inertial extensions of this paradigm including LIO-mapping [21], LINS [22], and LIO-SAM [19] added IMU fusion for high-rate motion prediction and improved robustness with LIO-SAM’s factor-graph back-end becoming a widely used baseline due to its native support for loop closure. Additionally, FAST-LIO [23] and FAST-LIO2 [24] marked a shift away from explicit feature extraction, instead registering points directly against the map within an iterated Kalman filter, foreshadowing the fully direct approach adopted in this work.

Direct LiDAR odometry (DLO) [25] and Direct LiDAR-Inertial Odometry (DLIO) [26] dispense with feature extraction entirely, registering point clouds directly against an adaptively maintained keyframe submap via GICP. DLO introduces adaptive keyframing to bound map growth and computation while preserving registration accuracy at the LiDAR frame rate. DLIO extends this framework with IMU preintegration for high-rate pose prediction and, critically, continuous-time per-point motion correction, which corrects for intra-scan distortion more accurately than whole-scan deskewing under fast or aggressive motion. Moreover, subsequent methods have continued to develop this direct paradigm: Point-LIO [27] processes measurements at native per-point rate for high-bandwidth motion, Faster-LIO [28] accelerates map queries via incremental voxel structures, and VoxelMap [29] replaces point-based maps with probabilistic voxel-plane representations. These successors, together with pose-graph extensions such as D-LIOM [30], primarily target loop closure and further efficiency gains rather than revisiting the core direct-registration formulation established by DLO/DLIO. In this paper, we use the direct LiDAR odometry [25] for simultaneous localization and mapping with Spot.

## 2. Materials and Methods

The materials and software that are used in the methods are as follows: Boston Dynamics Spot, Boston Dynamics, Waltham, MA, USA; Velodyne VLP-16, Velodyne Lidar, San Jose, CA, USA; FARO Focus X330, Faro Technologies, Berwyn, PA, USA; Logitech HD Webcam C270, Logitech International S.A., Lausanne, Switzerland; Unity version 2022.3.20f1, Unity Technologies, San Francisco, CA, USA.

In this section, we will describe the 3D localization of the Boston Dynamics robotic dog (which will be called Spot for the remainder of this paper), Section 2.1, and provide a brief summary of image keypoint tracking and frame matching to the localization data, as the prerequisites for obtaining 3D–2D matches for camera calibration (Section 2.2). Then, we will present a method to construct a virtual calibration pattern, a single-pass planar algorithm for focal length estimation, and a single-pass planar algorithm for extrinsic camera parameter estimation (Section 2.3).

It is worth noting that our methodology assumes that differences in focal length and camera orientation, as well as other intrinsic parameters—the principal point, aspect ratio, and lens distortion—will not change after manufacturing, do not have a huge impact for localizing persons in our applications, and are either centered, equal, or negligible, respectively, or they have been estimated offline in advance.

**Figure 1:**
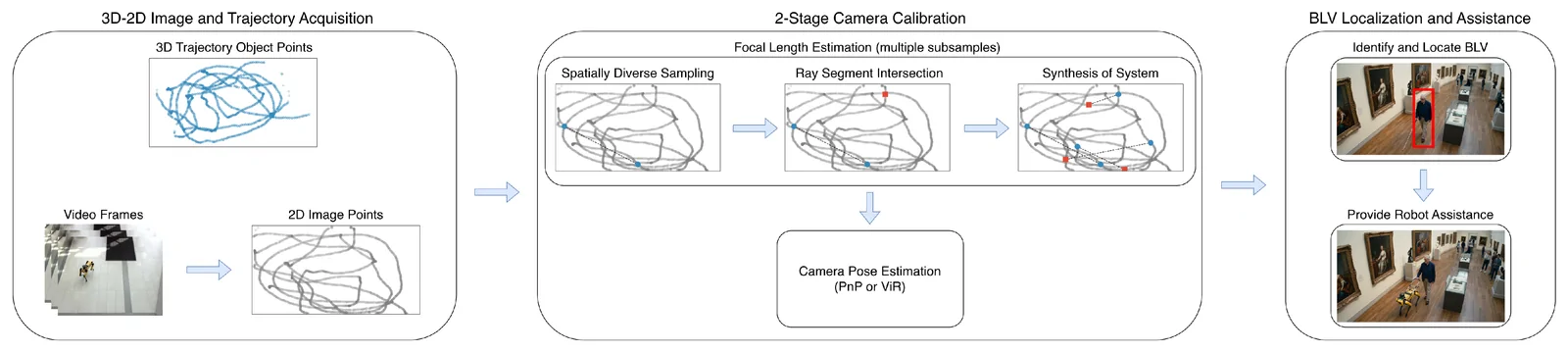
The proposed system diagram, with three modules: Robot Image and Trajectory Data Acquisition, Single-Pass 2-Stage Planar Calibration, and Assistive Framework Integration for BLV Localization (Future Work).

Figure 1 shows the proposed system architecture. We first acquire the robot dog’s trajectory along with the images of its trajectory. We then perform a 2-stage planar calibration, first estimating the focal length of the camera, then estimating the extrinsic camera parameters with either a planar PnP algorithm or one based on “virtual rectangles”. Once the camera is calibrated, we can locate individuals, relative to the robot dog’s global coordinate frame with the calibrated camera, so that the robot dog may provide assistance.

### 2.1. Obtaining the Robot’s 3D Trajectory Data and the Ground-Truth of the Camera Pose

#### 2.1.1. Real-Time 3D Trajectory Acquisition

In this study, we instrument Spot to gather its 3D trajectory data, including both location and orientation information, in real-time. Spot has a built-in vision-odometry-based self-localization module known as the Autowalk service [5]. According to our previous evaluation [31], the self-localization error of this approach drastically increases over time (err = 0.055 m, distance ⩾ 50 m; err = 0.141 m, distance ⩾ 100 m; err = 0.403 m, distance ⩾ 200 m). Therefore, to improve the quality of Spot’s trajectory data, we use direct LiDAR odometry [25], a simultaneous localization and mapping (SLAM) approach that leverages an inertial measuring unit (IMU) and a laser scanner to compute the 3D trajectory with an accuracy of 0.0216 m when the distance ⩾ 650 m in an outdoor environment [26]. Additionally, the direct LiDAR odometry approach does not require setting up any robot-standard fiducial markers in the environment to anchor the robot’s trajectory to the global coordinate frame [32].

#### 2.1.2. Offline Preparations: Ground-Truth Mapping

Our experiment is conducted in the lobby of the Rutgers Richard Weeks Hall (RWH) building. The primary purpose of this first offline stage is to map the environment and establish the ground-truth baseline for evaluating our calibration pipeline in a real-world scenario. A Logitech HD Webcam C270 target camera [33] is installed on the second floor pointing to the first floor (lobby). We place a sticker to mark an open ground area in the lobby (where the robot will walk) and adjust the pose of the camera to align the image center with the sticker. Because the Spot-carried Velodyne VLP-16 laser scanner has a limited vertical field of view (FOV), the target camera is barely visible to the robot. Therefore, once the camera pose is fixed, we employ a static laser scanner (FARO Focus X330) to collect a high-definition 3D point cloud of our testbed. This static scan is sufficiently dense to annotate the ground-truth location (lens) and pose (lens-to-mark) for the target camera in global coordinate frames, which is necessary to evaluate the accuracy of our calibration algorithm.

#### 2.1.3. Ground-Truth Coordinate Alignment

**Figure 2:**
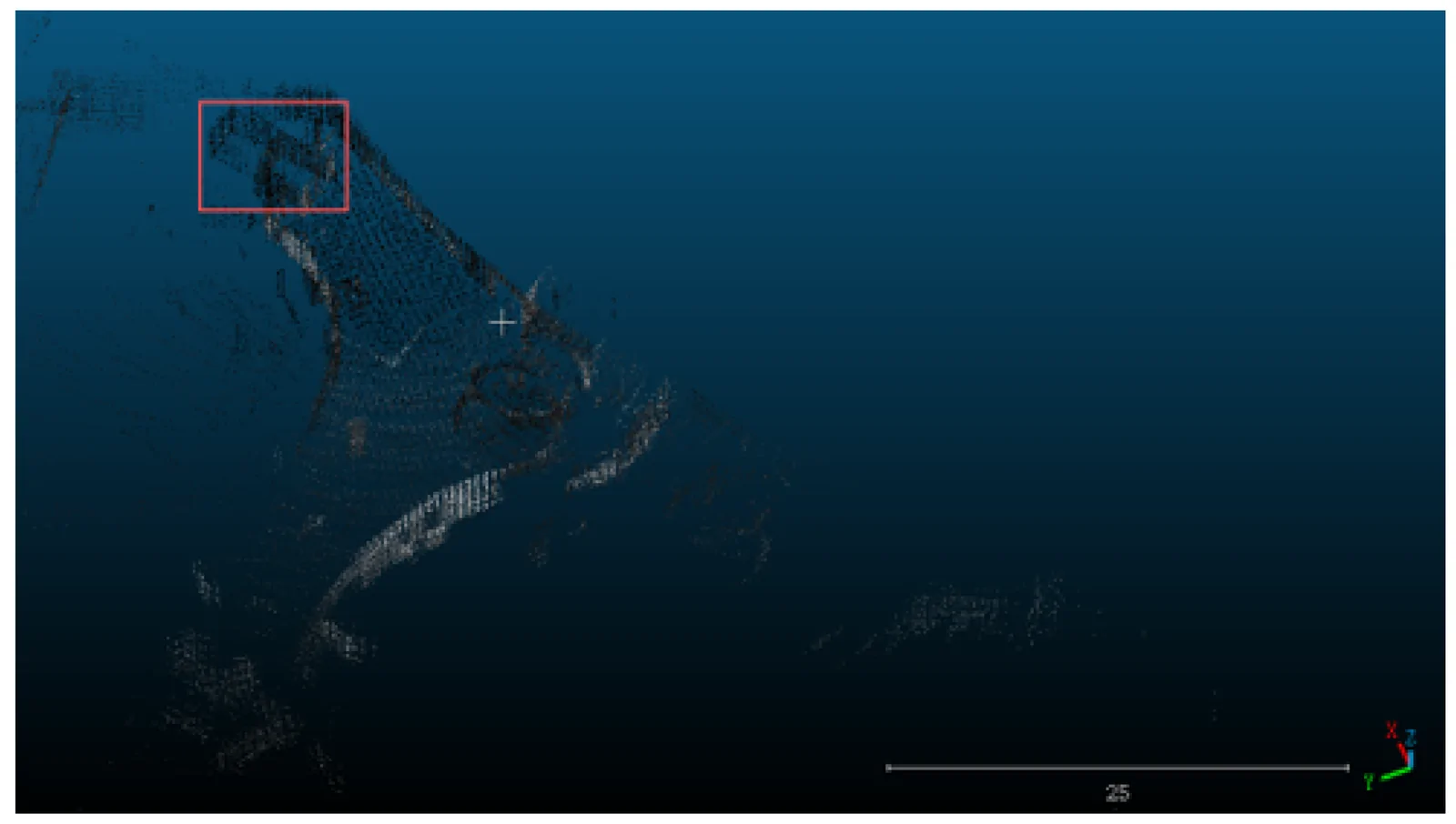
The Spot-mounted SLAM-generated point cloud. Red box indicates the region of the red door.

**Figure 3:**
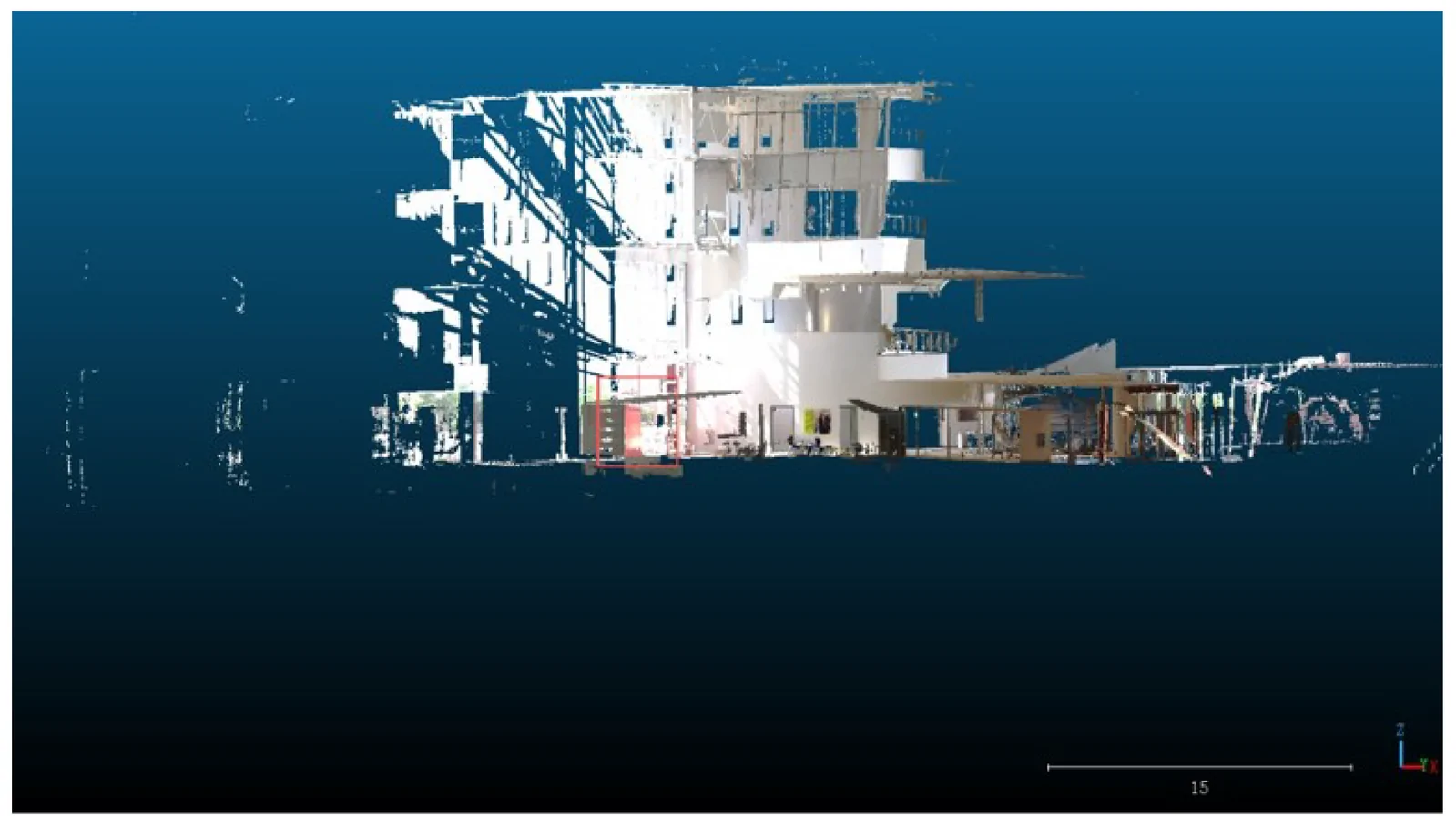
The higher-resolution static laser scan. Red box indicates region of the red door.

Since the SLAM-generated point cloud (Figure 2) and the static laser scan (Figure 3) operate in different local coordinate systems, a spatial registration is required to unify them. It is important to note that this spatial registration—including the extraction of planes and the alignment of the coordinate systems—is performed strictly to acquire the ground-truth measurements for validation, and is not a part of the proposed automated calibration pipeline.

The red boxes mark the location of the same reference object (the red door) in these two different point clouds. To align them, we extract three distinct corresponding planar surfaces—the floor, a wall, and a door—from both point clouds. These three planes are deliberately selected because their normal vectors are non-parallel and pairwise intersecting, providing a fully constrained geometric relationship. By computing and aligning the normal vectors of these corresponding planes, we derive the rigid transformation matrix between the two spaces. The target camera and the sticker on the floor in the static laser scan coordinate system are rotated with

(1) $$R=\left[\begin{array}{ccc} -0.95614518 & -0.29284537 & 0.00528953\\ 0.29285947 & -0.95615317 & 0.00210681\\ 0.00444063 & 0.00356351 & 0.99998379 \end{array}\right],$$

and are translated with

(2) $$t={\left[\begin{array}{ccc} -1.37806505 & -1.37945869 & 11.08689680 \end{array}\right]}^{T},$$

to the SLAM coordinate system. Once the spatial coordinates are unified, we calculate the 6-DoF pose of the target camera based on geometric constraints. Because the camera lens was physically oriented to point directly at the floor sticker, the directional vector originating from the camera’s optical center to the sticker mathematically defines the camera’s optical axis (the local Z-axis). Furthermore, during the hardware installation, the camera is carefully leveled to ensure its local X-axis remained strictly horizontal. With the Z-axis uniquely defined by the line-of-sight vector and the X-axis completely constrained to the horizontal plane, the camera’s Y-axis is derived via the cross product, fully determining its rotation matrix.

**Figure 4:**
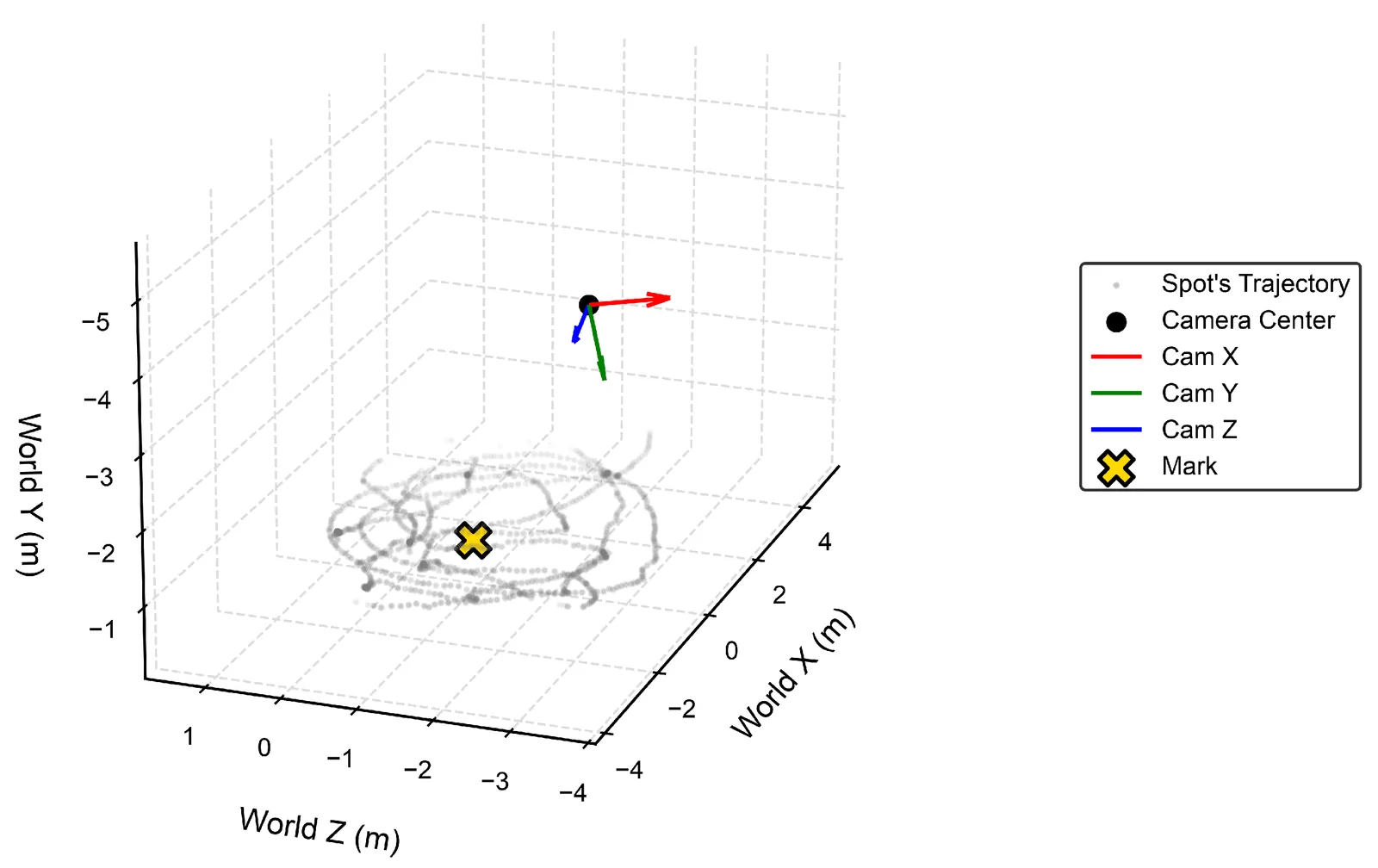
Camera pose visualization with Spot’s trajectory in global coordinate frame.

Subsequently, based on the SLAM coordinate system and its data points, we transform the entire coordinate system from a right-handed system with the Z-axis pointing upwards to a right-handed system with the Y-axis pointing downwards to facilitate our subsequent calibration algorithm. Specifically, this process involves swapping the Y and Z coordinates of all points in the SLAM coordinate system and then inverting the Y coordinates. Combining this orientation with the transformed location yields the ground-truth extrinsic camera parameters (Table 1) of the camera within the SLAM framework. We illustrate the camera pose with Spot’s trajectory within the global coordinate frame in Figure 4.

#### 2.1.4. Online Phase: 3D Trajectory Acquisition

In the online stage, Spot randomly walks around the lobby within the target camera view. Its trajectory data is collected at a frame rate of 10 fps (capped by the Velodyne VLP-16 laser scanner mounted on Spot’s back) while the target camera records at a resolution of 640 (width) by 480 (height) at 30 fps. Spot’s trajectory and the SLAM-mapped environment are recorded with the Robot Operating System (ROS), while the video from the target camera is recorded separately. Data from both sources are captured at different frame rates but are synchronized using timestamps.

### 2.2. Image Point Acquisition

#### 2.2.1. Offline Phase: Object Detection Model Training

**Figure 5:**
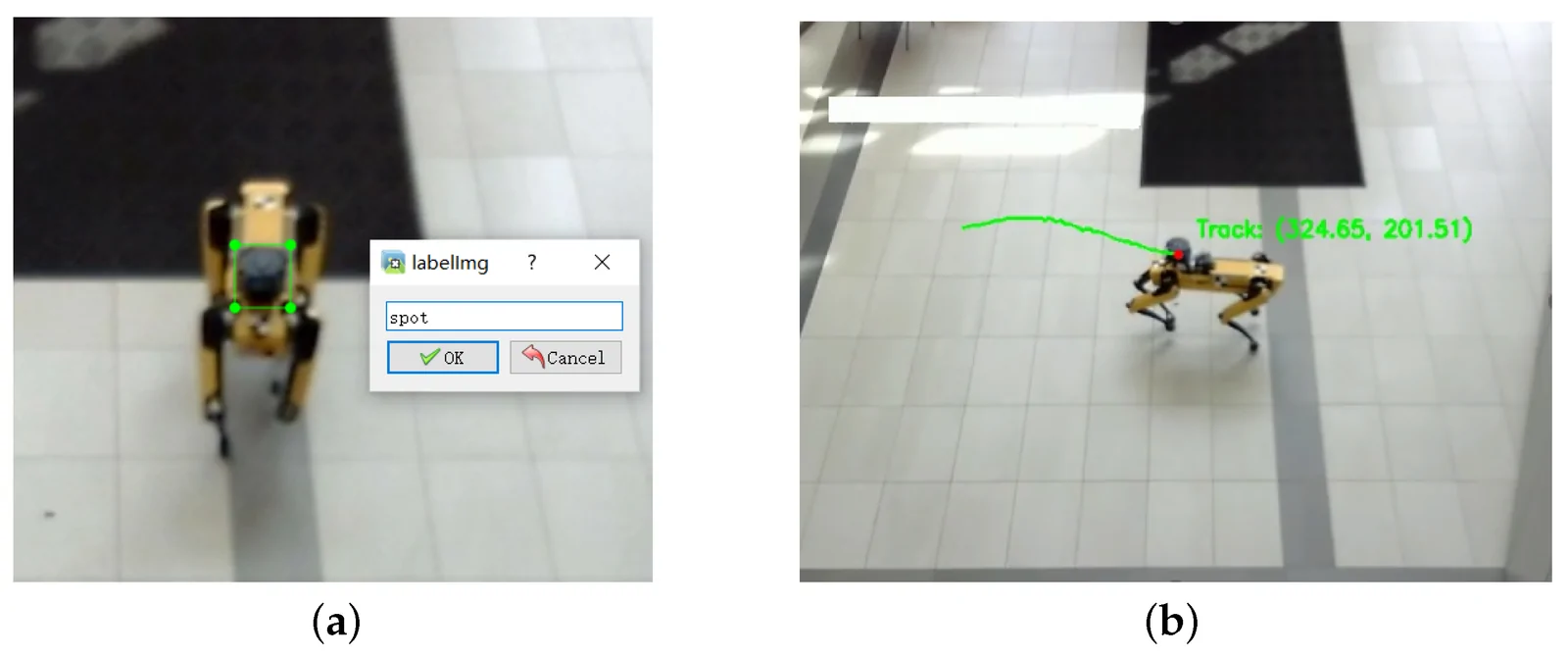
(**a**) Image labels applied for Spot’s keypoint, green bounding box is keypoint label applied to image; (**b**) keypoint tracking of Spot’s keypoint, green line is keypoint trajectory and red dot is current keypoint.

To obtain the 2D image coordinates of Spot’s keypoint, we utilize an object detection model, YOLOv8 [14], to automatically localize Spot’s LiDAR sensor in each image frame. We use the LiDAR sensor as a natural “calibration target” since it is always visible and easy to detect as long as the robot is within the camera’s field of view. To adapt the model to our specific setup for an accurate calibration target detection, we manually annotate the bounding boxes of the LiDAR sensor across several hundred images to construct a custom training dataset (Figure 5a).

#### 2.2.2. Online Phase: Image Point Acquisition

After training, the model can accurately output the bounding box of the sensor in continuous frames. We then extract the geometric center of each detected rectangle and convert it into pixel coordinates (Figure 5b). This straightforward tracking process successfully provides stable 2D observations, which are subsequently paired with the real-world 3D coordinates recorded by the SLAM device for the calibration process.

Meanwhile, the corresponding global 3D coordinates of Spot are obtained directly from the synchronized LiDAR measurements. By matching the detected image positions with the recorded LiDAR coordinates through timestamps, we establish 3D–2D correspondences for every valid frame. These correspondences serve as the foundation for the subsequent calibration algorithm, enabling the estimation of the focal length and extrinsic parameters. After verifying the consistency and accuracy of the matched data, the preprocessing stage is completed, yielding a high-quality dataset for the calibration experiments.

### 2.3. Method for Planar Camera Calibration

We propose a 2-stage method for camera calibration. First, we estimate the focal length of the camera with a RANSAC-like [34] methodology, where we acquire multiple subsets of “object points” from Spot’s trajectory in the global coordinate frame and their corresponding image points on the image plane. From each subset of points, we estimate a focal length. The mean of the estimated focal lengths from these subsets of points is then utilized as the final focal length estimate.

In the second stage, we estimate the extrinsic camera parameters with each subset of points, along with the final focal length estimate obtained in the first stage, and calculate the mean reprojection error from the estimated extrinsic camera parameters and final focal length. We then select extrinsic camera parameters that minimize the reprojection error. Two planar algorithms are compared in the second stage: our proposed “Virtual Rectangle” (ViR) algorithm, and the standard planar PnP algorithm [6].

In this section, we detail the selection process of the global points with a three-step algorithm in automated virtual rectangle construction (Section 2.3.1). Then, we describe the two-stage planar calibration approach (Section 2.3.2): the calibration of the camera focal length from those points (Stage I), and the calibration of the extrinsic parameters of the camera relative to the global frame (Stage II).

#### 2.3.1. Automated Virtual Geometric Structure Construction: A Three-Step Algorithm

This section details the algorithm used to extract a geometrically consistent “virtual calibration pattern” from the unstructured trajectory of Spot. We programmatically identify points within the robot’s odometry history that form rigid geometric relationships (parallelism and orthogonality) with sufficient spatial separation to ensure numerical stability.

To compute the homography and recover camera parameters, we require a set of four global points that form a known geometric structure (2 pairs of parallel lines—thus, a “virtual rectangle”) on the ground plane. However, the robot’s trajectory $T=\{P_{1},\ldots ,P_{N}\}$ is effectively random. Therefore, we develop a three-step algorithm to mine T for point configurations that satisfy strict distance and geometric constraints in both the 3D ground plane that the robot walks on and their corresponding 2D points on images. We assume the robotic trajectory is on the ground plane, which can be evaluated and selected after Spot obtains its 3D trajectory.

##### Step 1: Spatially Diverse Sampling

To avoid degenerate solutions where calibration points are clustered too closely, we first define a selection function that enforces a minimum Euclidean separation $\delta_{min}$ on the 3D plane. Let S be the set of selected indices. We iteratively select a candidate point $P_{c}$ from T and accept it into S only if it satisfies the distance constraint with respect to all previously selected points:

(3) $$\forall P_{k}\in S,\;{∥P_{c}-P_{k}∥}_{2}\geq \delta_{min}$$

**Figure 6:**
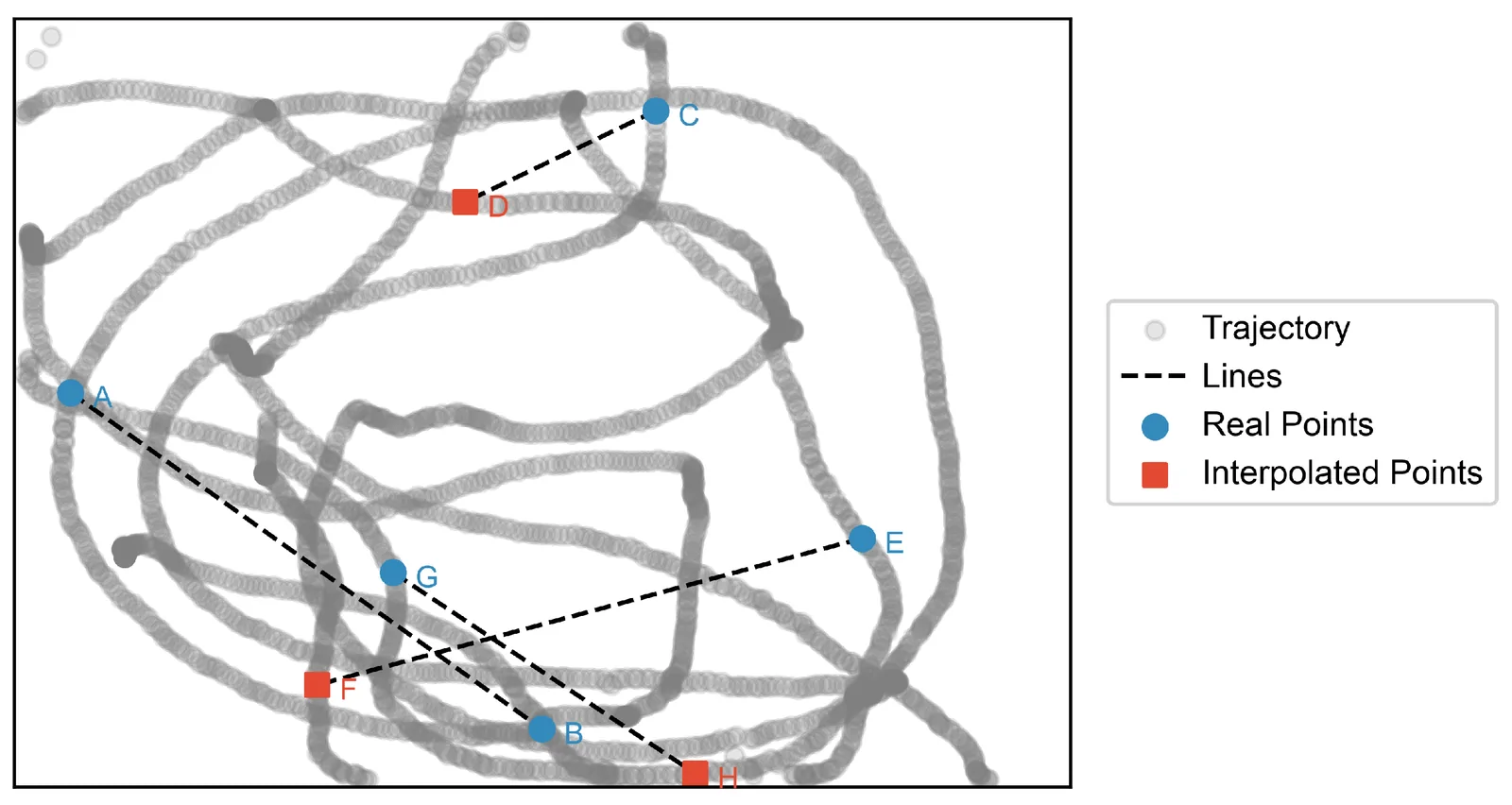
Eight-point system on the image plane, with five real points (red) and three interpolated virtual points (blue). AB is parallel to GH and CD is parallel to EF.

This ensures that the baseline for our geometric calculations spans a sufficient physical distance (based on the trajectory range) to minimize the impact of sensor noise. In this phase we select three points (A,B,C in Figure 6) for S satisfying the condition.

##### Step 2: Ray-Segment Intersection with Distance Filtering

A core component of our algorithm is the ability to “cast” a geometric ray from a known point $P_{start}$ along a direction vector v and find where it intersects the robot’s past trajectory. Any two points along Spot’s trajectory (A,B) can form a line, $\vec{AB}$, and any other point on the trajectory, *C*, can be the starting point of a line perpendicular to $\vec{AB}$. We select an interpolated “virtual” point, *D*. The trajectory of Spot is discrete, so we treat it as a piecewise linear path connecting consecutive points $P_{i}$ and $P_{i+1}$. We solve for the intersection point *D* by iteratively scanning *i* until the trajectory segment satisfies

(4) $$D=P_{i}+u\left(P_{i+1}-P_{i}\right)$$

for 0≤u≤1, and the resulting point maintains the minimum separation distance from the source:

(5) $$∥D-P_{i}{∥}_{2}\geq \delta_{min}$$

We add *D* to S. The corresponding image point is interpolated from *u* such that

(6) $$D_{img}=p_{i}+u\left(p_{i+1}-p_{i}\right),$$

where $p_{i}$ is the corresponding image point of the 3D point $P_{i}$.

We would like to note here that we tried to revise the focal length estimation with a new *u* calculated from a supposedly more accurate interpolation using the homography. However, it did not produce better results in focal length estimation compared to a simple affine approach while increasing computational time.

##### Step 3: Synthesis of Orthogonal Systems

Finally, we construct a complete geometric system (points E,F,G,H) so that the rays extending from the vectors $\vec{AB},\vec{CD},\vec{EF},\vec{GH}$ intersect to form a rectangle. Given two real baseline points *A* and *B*, we compute the perpendicular vector $\vec{EF}⊥\vec{AB}$ by finding a point *E* that satisfies the constraint, such that

(7) $${\forall P\in S,\;∥P-E∥}_{2}\geq \delta_{min}$$

Then we select another interpolated point *F*. We repeat this process to find points *G* and *H* from points *C* and *D*. This process retrieves 2 pairs of parallel lines perpendicular to each other on the floor using Spot’s trajectory (Figure 6).

#### 2.3.2. Planar Camera Calibration: A Two-Stage Approach

##### Stage I: Focal Length Calibration

We utilize the virtual rectangle derived from the eight-point system (five real and three virtual) to calculate two vanishing points from the projected image points of the two perpendicular pairs of parallel lines in order to calibrate the camera’s focal length.

In projective geometry, parallel lines in 3D space intersect at a single “vanishing point” (VP) on the 2D image plane. We have two sets of parallel lines: $\left(\bar{AB},\bar{GH}\right)$ and $\left(\bar{CD},\bar{EF}\right)$. To compute the vanishing point v for a pair of parallel segments defined by image points p, we utilize homogeneous coordinates where $p={\left[u,v,1\right]}^{T}$. The line l connecting two points $p_{1}$ and $p_{2}$ is given by the cross product $l=p_{1}\times p_{2}$. The vanishing point v, which represents the intersection of two image lines $l_{1}$ and $l_{2}$, is computed as $v=l_{1}\times l_{2}$.

We then normalize v by its third component to recover the Cartesian coordinates in the image plane. This process yields two orthogonal vanishing points, $v_{1}$ and $v_{2}$, where $v_{i}={\left(x_{i},y_{i},f\right)}^{T},i=1,2$, and f is the unknown focal length of the camera.

Recovering the focal length relies on the orthogonality constraint. Since the lines are orthogonal in 3D space, the vectors extending from the camera’s optical center to the two vanishing points must also be orthogonal. We approximate the principal point $\left(c_{x},c_{y}\right)$ as the geometric center of the image (width W/2, height H/2). We then shift the vanishing points to this centered coordinate system:

(8) $$v_{i}^{\prime }=v_{i}-{\left[c_{x},c_{y},0\right]}^{T}$$

where $v_{i}^{\prime }=\left(x_{i}^{\prime },y_{i}^{\prime },f\right),i=1,2$. Since $v_{1}^{\prime }\cdot v_{2}^{\prime }=0$, the focal length *f* is now calculated from the following:

(9) $$f=\sqrt{-(x_{1}^{\prime }x_{2}^{\prime }+y_{1}^{\prime }y_{2}^{\prime })}$$

For a valid solution, the term under the square root must be positive, which implies that the dot product of the shifted vanishing points in the 2D image space must be negative. Geometrically, this confirms that the vanishing points lie on opposite sides of the principal point, consistent with the projection of orthogonal axes. If the dot product is non-negative, the configuration is degenerate (e.g., one or both of the vanishing points are in or close to infinite), the sample is discarded and we find a new eight-point system. Note that in finding the focal length, we assume the image center and the aspect ratio is known, which are typically in the middle of the captured image, and 1:1, respectively.

##### Stage II: Extrinsic Camera Calibration

For the extrinsic camera calibration, we test two separate algorithms: the planar PnP algorithm detailed in [6] and an algorithm utilizing the intermediate local planar rectangle generated above. Our Virtual Rectangle (ViR) algorithm is detailed below.

Since we establish the focal length (*f*) via vanishing points, we proceed to recover the full 6-DoF pose of the camera relative to the global frame while assuming a centered principal point, equal unit aspect ratio, and negligible lens distortion. To facilitate this, we first define an intermediate local coordinate system aligned with the geometry of the virtual rectangle constructed from the two pairs of parallel lines.

- Rotation and Translation Estimation—The transformation from the local rectangle to the camera frame is modeled as
  (10) $$P_{c}=RP_{l}+T$$
  Rotation, $R=[r_{1}\;r_{2}\;r_{3}]$, is constructed using unit vectors derived from vanishing points. To recover translation T and point depths $Z_{k}$, we substitute the five known “real” points $P_{l}$ (from the rectangle’s construction) and their image projections p into the perspective constraint
  (11) $$Z_{k}\bar{p}=K\left(RP_{l}+T\right),$$
  solving the resulting linear system, with K being the intrinsic camera parameter matrix.
- Final Global Transformation—We compute the camera pose ($R_{cg},T_{c}$) relative to the global environment by combining the camera-to-local pose with the known global-to-local rectangle transformation ($R_{gl},T_{g}$). Substituting the inverse relation $P_{l}=R_{gl}^{T}\left(P_{g}-T_{g}\right)$ into the camera equation yields the final calibration parameters:
  (12) $$R_{cg}=RR_{gl}^{T},\;T_{c}=T-R_{cg}T_{g}$$

### 2.4. Assistive Framework Integration

Our assistive framework is to utilize the estimated focal length and extrinsic camera parameters in order to estimate the position of a BLV individual within a camera network in Spot’s (global) coordinate frame so that Spot may locate and assist an individual by simply locating them in the camera network. As a person is detected in the image, by utilizing the same object detection model YOLOv8 [14] trained for persons (as well as genders), as in Section 2.2, so that we can obtain the person’s pixel location (u,v) with a height of *h* on the image plane, we can perform 3D localization by first estimating the distance of the individual relative to the camera:

(13) $$Z_{cam}=f\cdot \frac{H}{h},$$

where $Z_{cam}$ is the depth of the individual in meters relative to the camera’s coordinate frame, *f* is the estimated focal length in pixels, *H* is the individual’s height (e.g., an average height of a male or female person) in meters, and *h* is the individual’s height in pixels on the image plane. We can then find the location of the individual relative to the camera, $P_{cam}$, with

(14) $$X_{cam}=Z_{cam}\cdot \frac{u-c_{x}}{f};\;Y_{cam}=Z_{cam}\cdot \frac{v-c_{y}}{f};\;P_{cam}={\left[X_{cam},Y_{cam},Z_{cam}\right]}^{T},$$

where (u,v) is the pixel coordinates of the individual in the camera’s image plane and $\left(c_{x},c_{y}\right)$ is the principle point of the camera.

With the estimated rotation, $R_{cg}$, and translation, $T_{c}$, of the camera in Spot’s global coordinate frame, we can estimate the BLV individual’s position in the global coordinate system as measured by Spot, $P_{global}$, with

(15) $$P_{global}=R_{cg}P_{cam}+T_{c}.$$

## 3. Results

We utilize four metrics to determine the efficacy of our methodology:

- Focal length error—The difference between the estimated focal length (pixels) and ground-truth focal length (pixels).
- Reprojection error—The mean Euclidean distance (pixels) of the reprojected image points and their corresponding original image points.
- Rotational error—The error, in degrees, between the estimated rotational matrix, $R_{est}$, and the ground-truth rotational matrix, $R_{gt}$, calculated by
  (16) $$\theta =\arccos\left(\frac{\mathrm{Tr}(R_{est}R_{gt}^{T})-1}{2}\right)$$
- Relative translation error—The error is calculated by the Euclidean distance of the estimated camera translation in the global frame from the ground-truth camera translation in the global frame divided by the mean camera distance from the trajectory’s object points.

Focal length error evaluates the accuracy of the estimation, reprojection error is utilized in evaluating the overall accuracy of camera parameter estimation, while rotational error and relative translation error determine the overall effectiveness of the extrinsic camera parameter estimation.

### 3.1. Real-World Results

In the following experiments, we perform 100 independent trials from the image and object point data we have collected in Section 2 to test the efficacy of our approach, and use 500 randomly sampled subsets of points (per trial) to calibrate the focal length and extrinsic camera parameters. We compare the extrinsic camera parameter estimates from our algorithms with the ground-truth camera pose obtained in Section 2.1. Additionally, the technical specifications [33] for the field of view (FOV) of the Logitech HD Webcam C270 is found to be inaccurate and the ground-truth focal length is calculated by manual measurement of a 1 m object covering the field of view at a distance of 1.5 m:

$$f=\left(img\text{\_}width/2\right)\frac{distance\text{\_}from\text{\_}object}{width\text{\_}of\text{\_}object/2}=320\;\mathrm{px}\left(\frac{1.5\;m}{0.5\;m}\right)=960\;\mathrm{px}.$$

#### 3.1.1. Real-World: Focal Length Estimation

**Figure 7:**
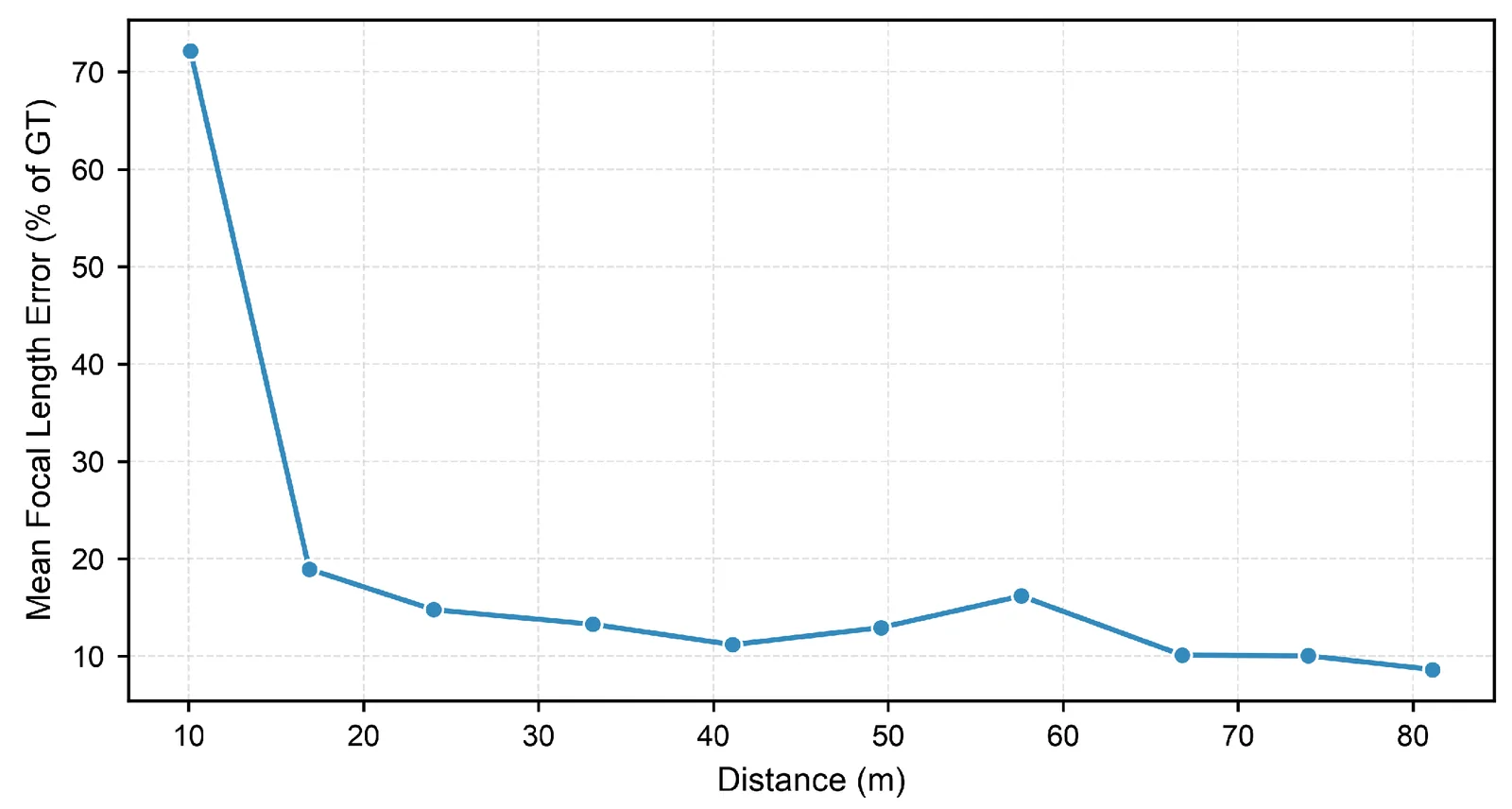
Focal length error compared with Spot’s trajectory distance.

In Figure 7, we conduct an experiment where we utilize the first *X*% of Spot’s trajectory, with *X* being from 10 to 100 in increments of 10. It is observed that the mean focal length estimation error across trials reduces with longer trajectories with the full trajectory producing the lowest mean focal length estimation error of 83 pixels. Table 2 illustrates the corresponding distance (in meters) and available points for sampling by increment. During our experiment, we controlled the total length of the robot trajectory (81.1 m) sufficiently smaller than the trajectory lengths in the experiments of the original DLO work to suppress the overall SLAM error (⩽0.02 m) caused by drifting over time.

**Figure 8:**
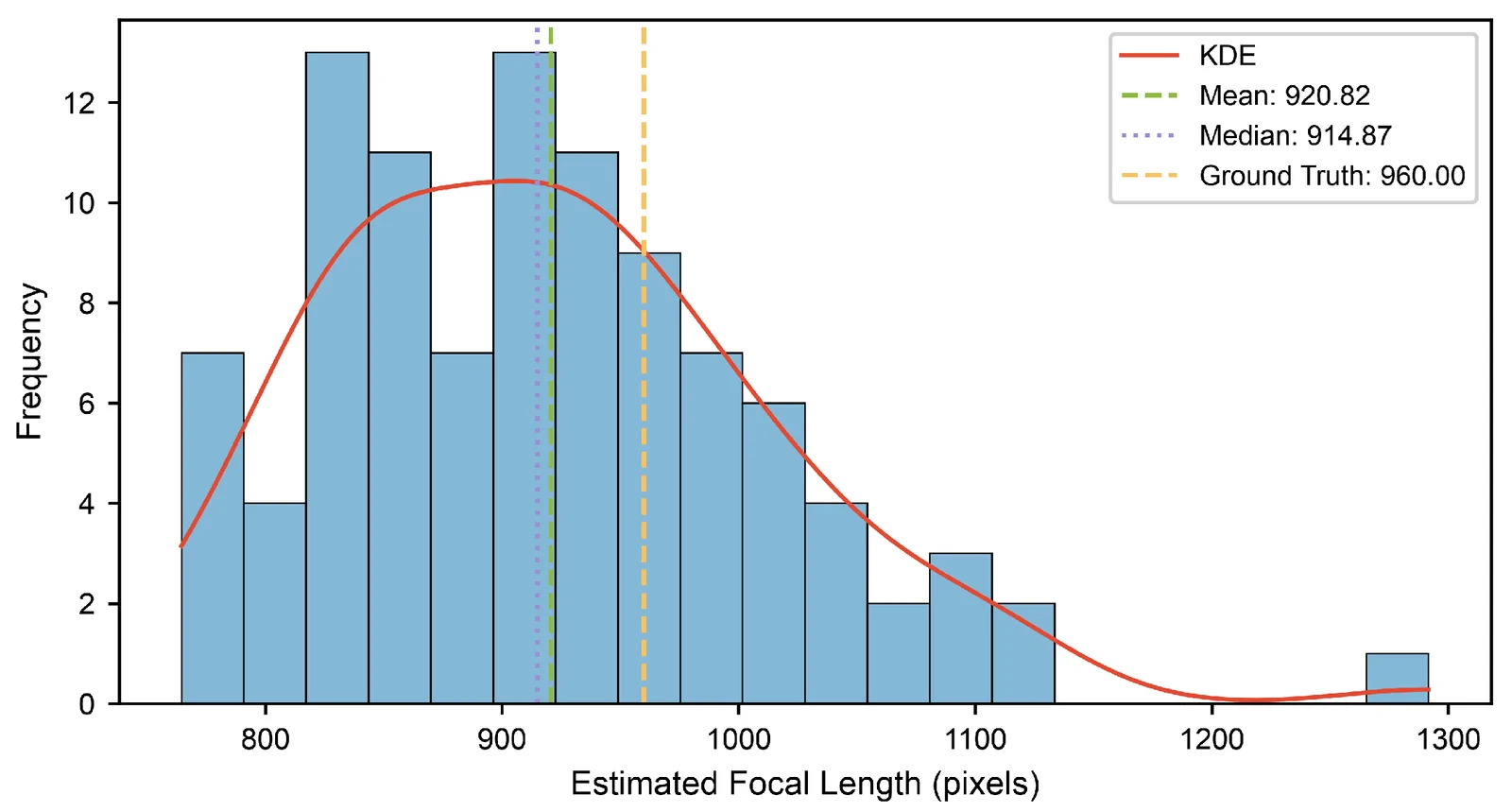
Histogram with kernel density estimate of focal length estimations.

We investigate the distribution of focal length estimates from the full trajectory in Figure 8. It is observed that the mean and median estimates are both less than 50 pixels from the calculated ground-truth, an error of less than 5%.

#### 3.1.2. Real-World: Reprojection Error

**Figure 9:**
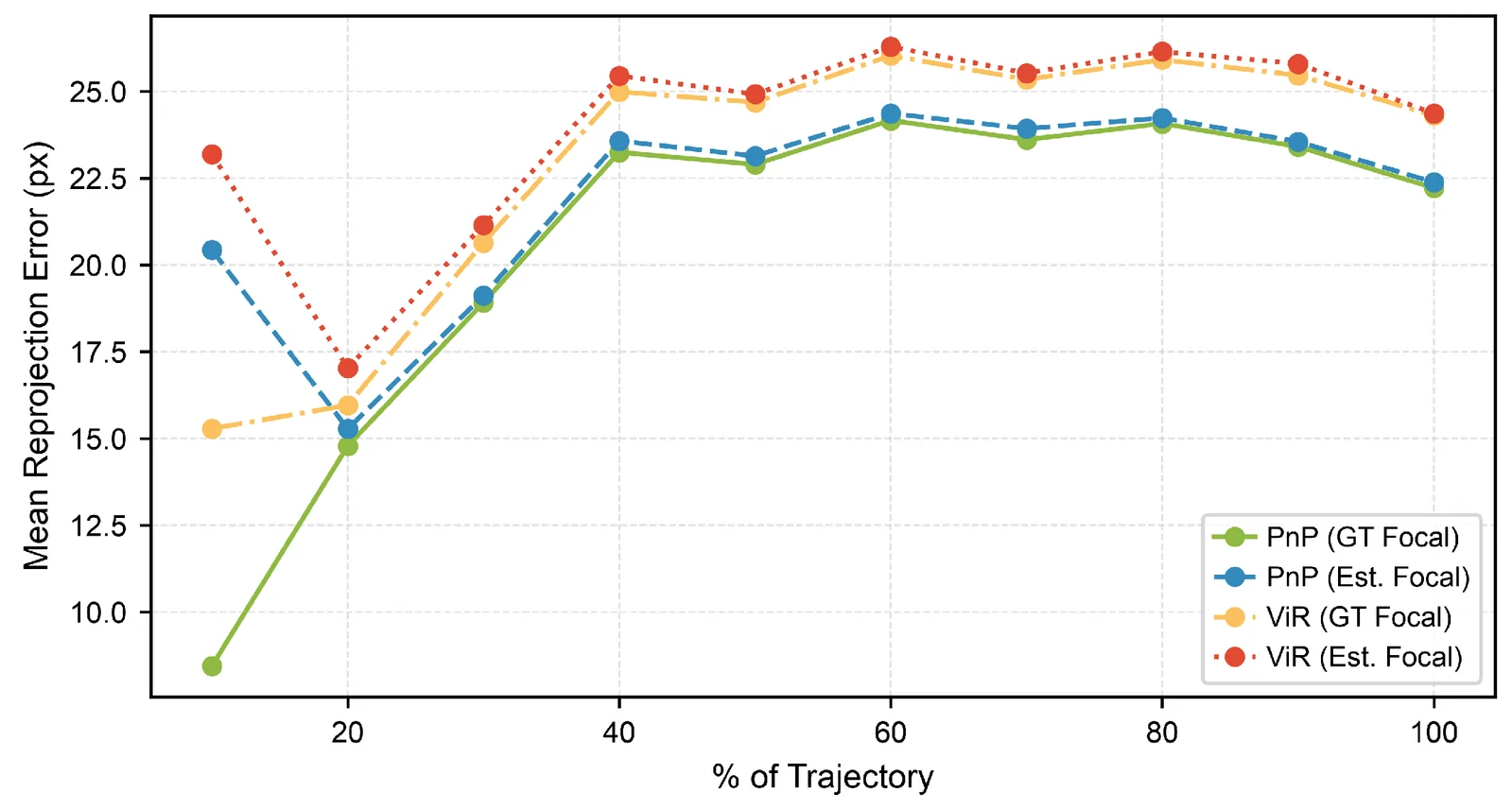
Mean reprojection error vs. the number of used points in trajectory with two extrinsic camera parameter estimation methods—PnP and ViR—using the estimated focal length against the ground-truth focal length.

In Figure 9, we compare the reprojection error of the estimated extrinsic camera parameters by the % of Spot’s trajectory and by methodology (PnP vs. our ViR algorithms). Additionally, we include a comparison of the estimated focal length and the ground-truth focal length that is utilized for extrinsic camera parameter estimation. The results appear to indicate that fewer available points for sampling of subsets produces lower reprojection errors (discussion in Section 4). The planar PnP algorithm also produces slightly better results relative to our algorithm (an error approximately 2 pixels less). Meanwhile, the ground-truth focal length that is used for extrinsic parameter estimation does not appear to produce a substantial improvement in comparison to the use of the focal length estimation produced by our algorithm. Overall, for most trajectory lengths, mean reprojection error is between 22 and 30 pixels.

#### 3.1.3. Real-World: Rotation Estimation Error

**Figure 10:**
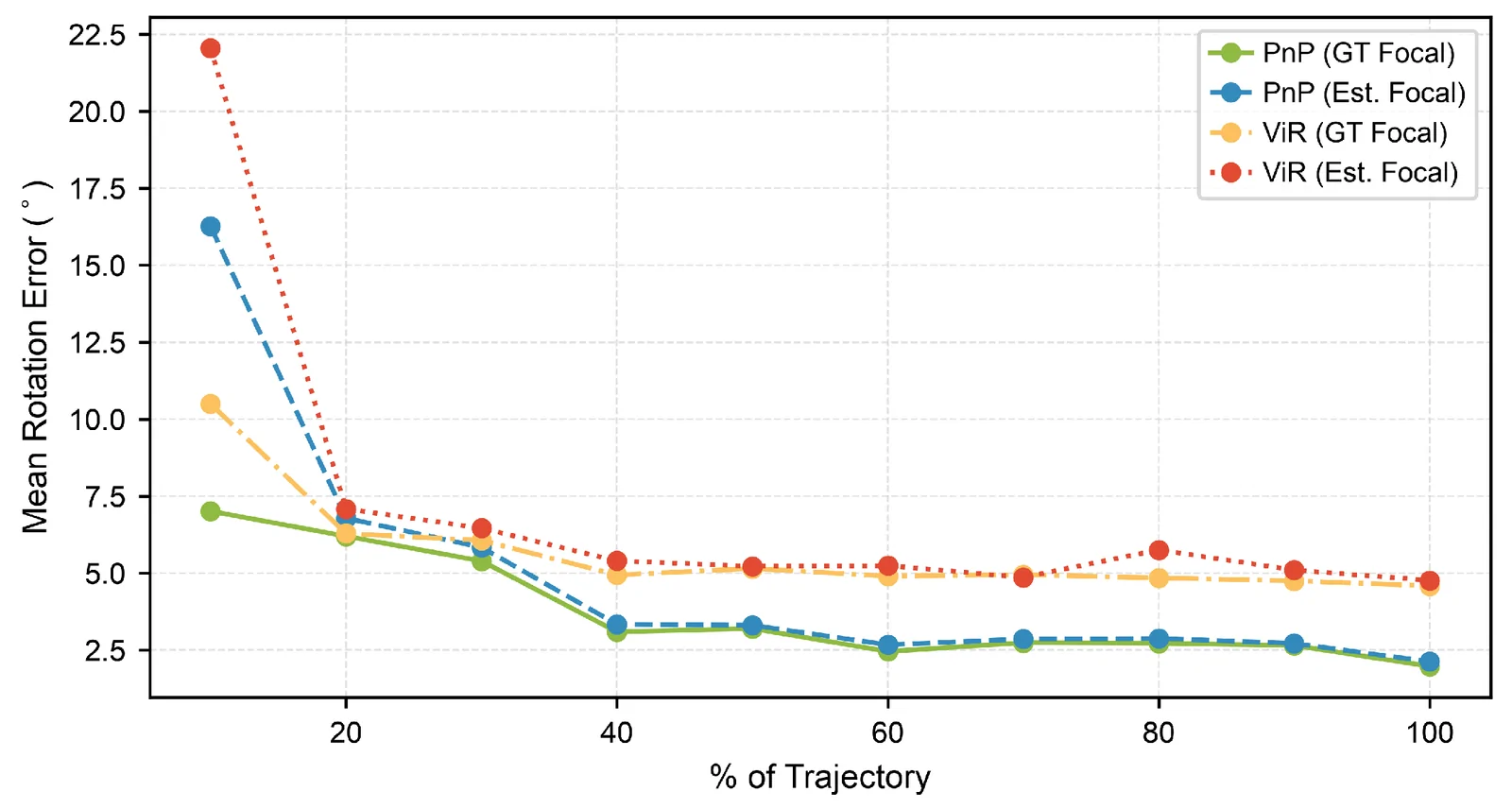
Mean rotational error vs. the number of used points in trajectory with two extrinsic camera parameter estimation methods—PnP and ViR—using the estimated focal length against the ground-truth focal length.

The results from the rotation estimation error analysis (Figure 10) illustrate a trend more consistent with the error trends in Figure 7. Increasing the number of points available for sampling decreases the amount of errors in estimating the rotation of the camera in the global coordinate frame. The planar PnP algorithm also estimates the camera rotation with roughly $2.5^{{}^{\circ}}$ less error in comparison to our ViR methodology. Again, utilizing the ground-truth focal length for extrinsic parameter estimation does not appear to affect the accuracy of rotation estimation of the camera. Across most trajectory lengths, rotation estimation error is below $5^{{}^{\circ}}$.

#### 3.1.4. Real-World: Translation Estimation Error

**Figure 11:**
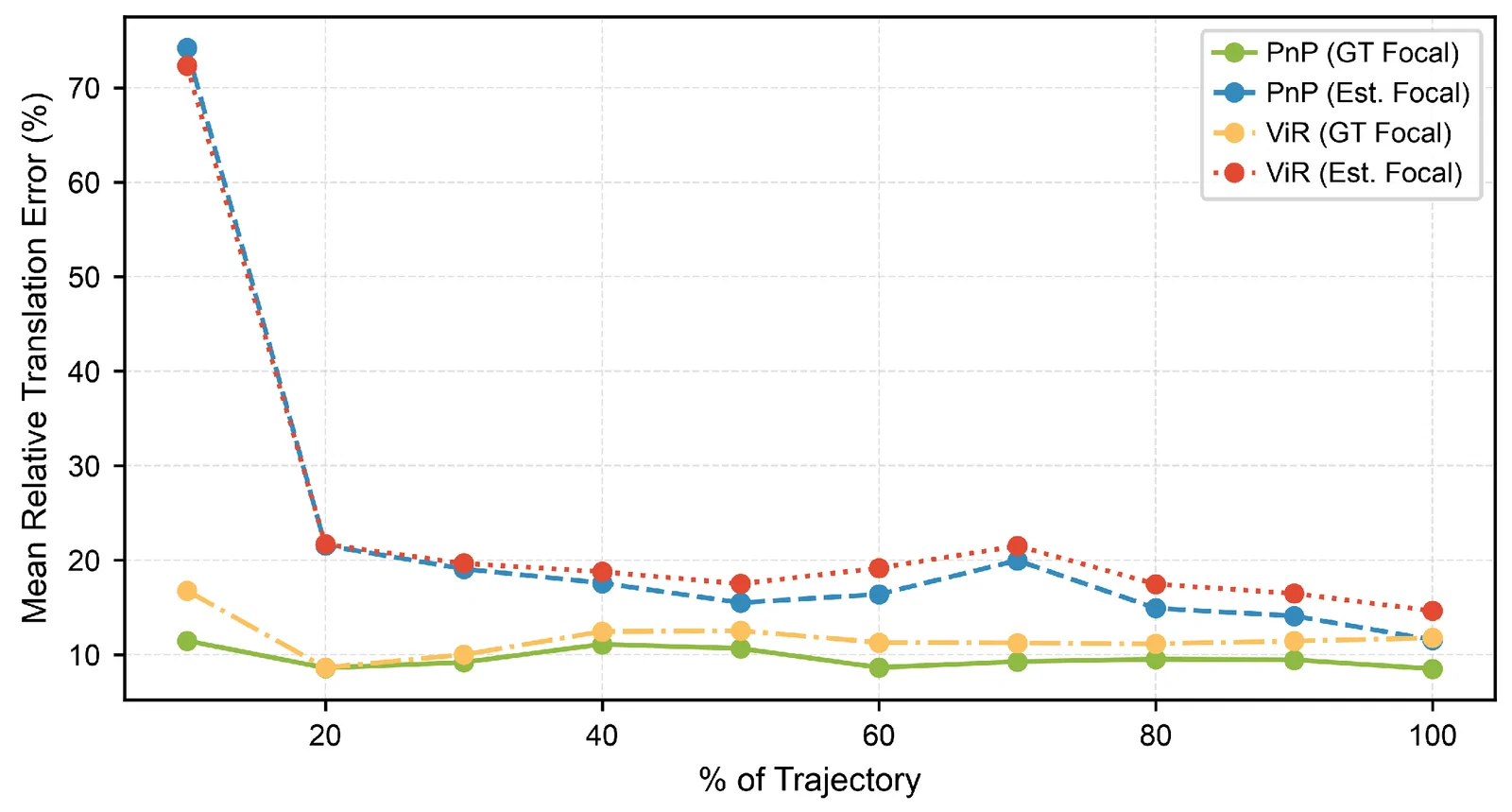
Relative translation error vs. the number of used points in trajectory with two extrinsic camera parameter estimation methods—PnP and ViR—using the estimated focal length against the ground-truth focal length.

Figure 11 further confirms the previous results: there appears to be a tendency for reduced relative translation error when there are more points to sample from. The planar PnP algorithm, once again, performs slightly better than our ViR algorithm (roughly 3 to 4 percentage points). However, we can observe that utilizing the ground-truth focal length in extrinsic parameter estimation produces better results across the board in translation estimation. Additionally, the mean relative translation error tends to be under 20% while trending towards 10% with increasing trajectory length.

### 3.2. Synthetic Results

**Figure 12:**
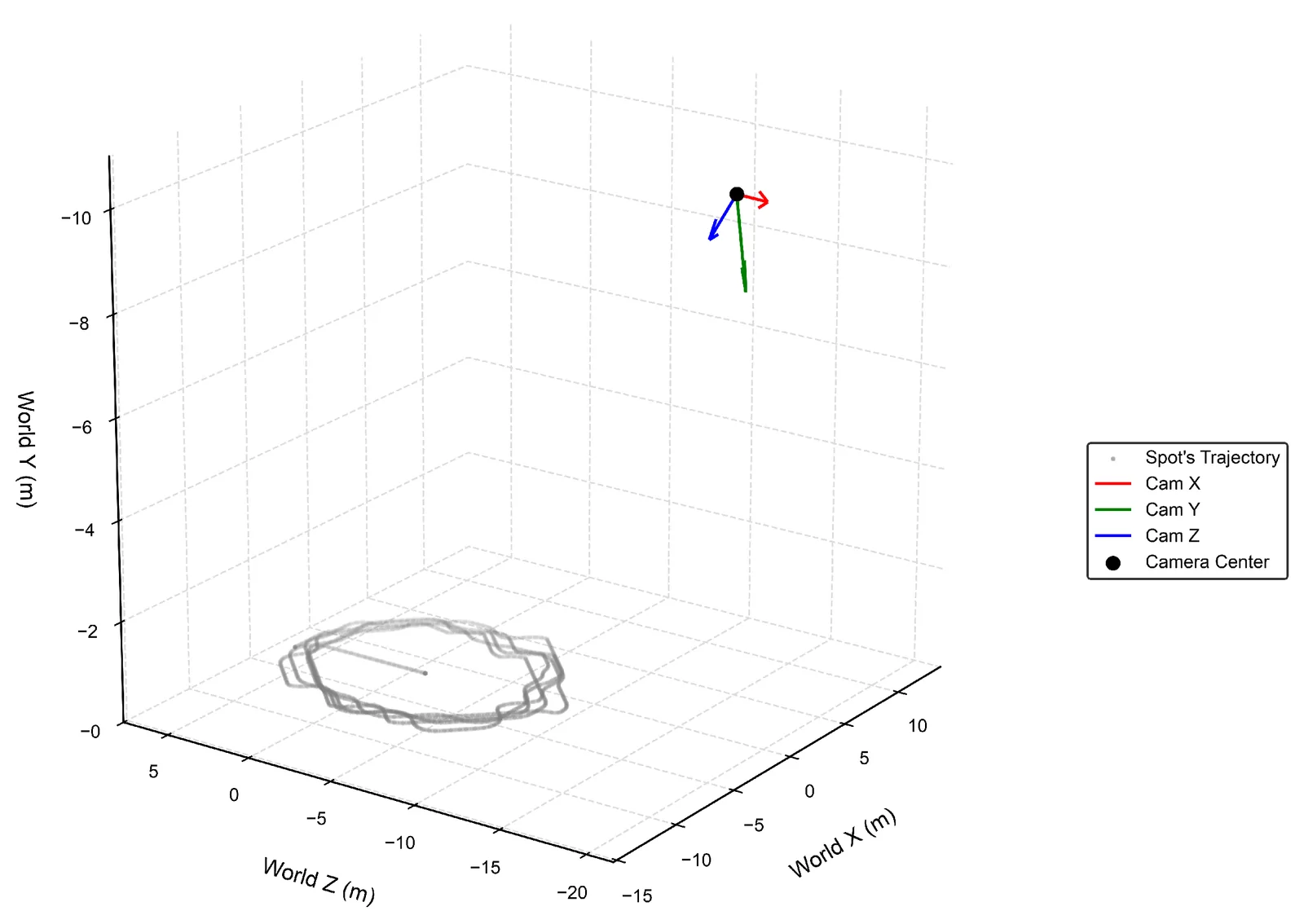
Virtual camera and synthetic Spot trajectory.

In order to test the accuracy and robustness of the algorithm with various situations—beyond a single trajectory, camera pose, and focal length—we utilize synthetic data generated by Unity [35]. The results from synthetic data also provide a reference point to estimating the inaccuracy of the real-world identification of Spot on the image plane and in the global coordinate frame. The virtual Spot traverses an approximately 16 m by 16 m floor space with one of the camera poses shown in Table 3 and visualized with respect to a virtual Spot trajectory in Figure 12. Image resolution is 1280 by 720 (pixels) and the focal length is 1430 (pixels). Additionally, the synthetic data trajectory contains over 12,000 image–object point pairs as opposed to the roughly 3000 image–object point pairs in the real-world data.

Gaussian noise is applied to each image point (u,v) with a mean magnitude of 0, 1, 2, 5, and 10 pixels as a method to emulate errors in the identification of the location of Spot on the image plane. From the real-world trajectory localization error, Gaussian noise is applied to each global object point (x,y,z) with a mean magnitude of 0, 0.05, 0.1, and 0.2 m as a way to simulate inaccuracy and sensor drift in Spot’s localization/trajectory. Additionally, in order to prevent results from occurring due to random chance, we, again, perform 100 independent trials for each combination of noise applied to the image points and the global object points.

#### 3.2.1. Synthetic: Focal Length Estimation

**Figure 13:**
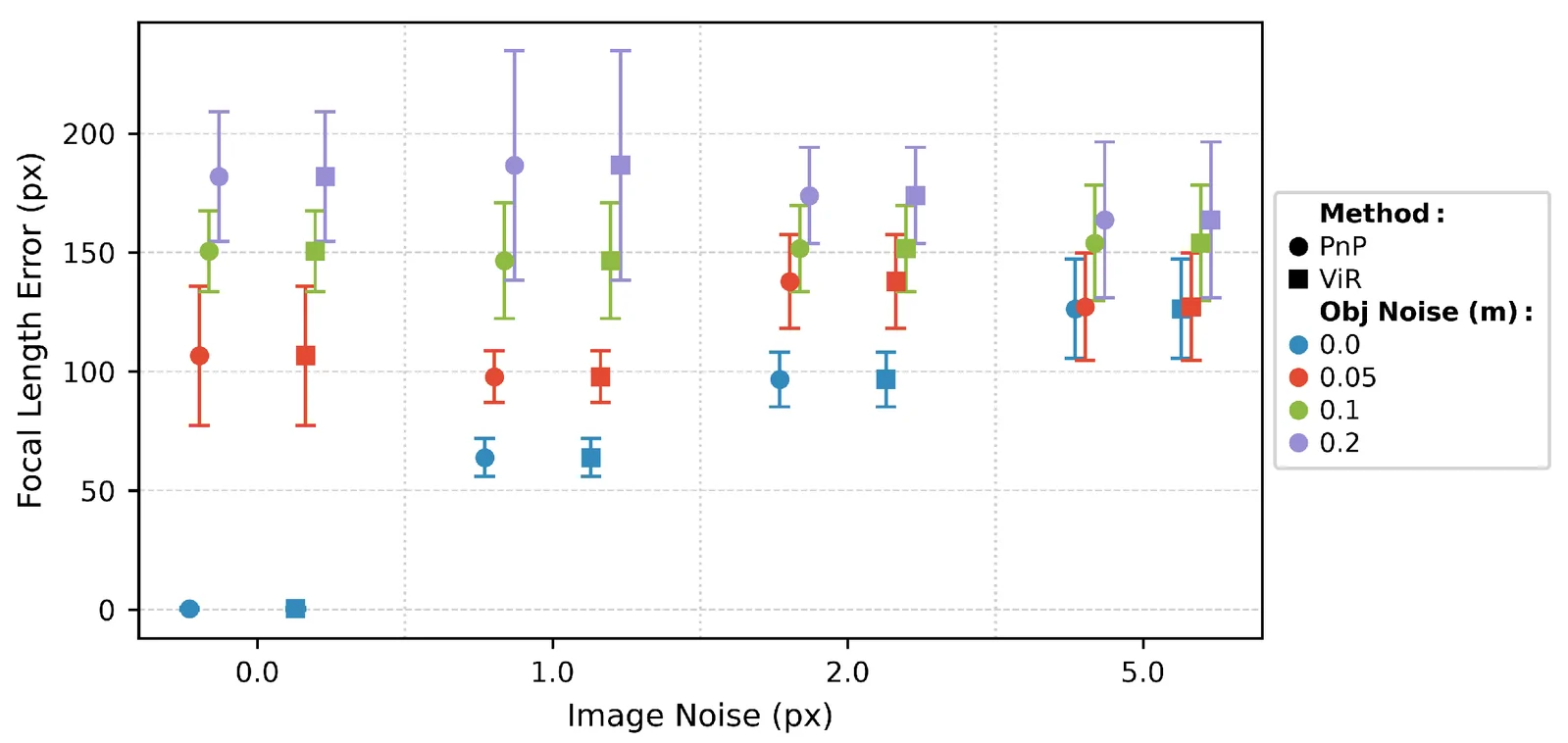
Focal length estimation error with 95% confidence interval.

In the results with synthetic data (Figure 13), image noise appears to have a more significant effect on focal length estimation. Error increases with increasing object noise. These results are fairly consistent with the real-world data as low object noise and around 1 to 2 pixels of image noise produce around 10% of focal length estimation error, which is similar to the mean focal length estimation error of the longer trajectories.

#### 3.2.2. Synthetic: Reprojection Error

**Figure 14:**
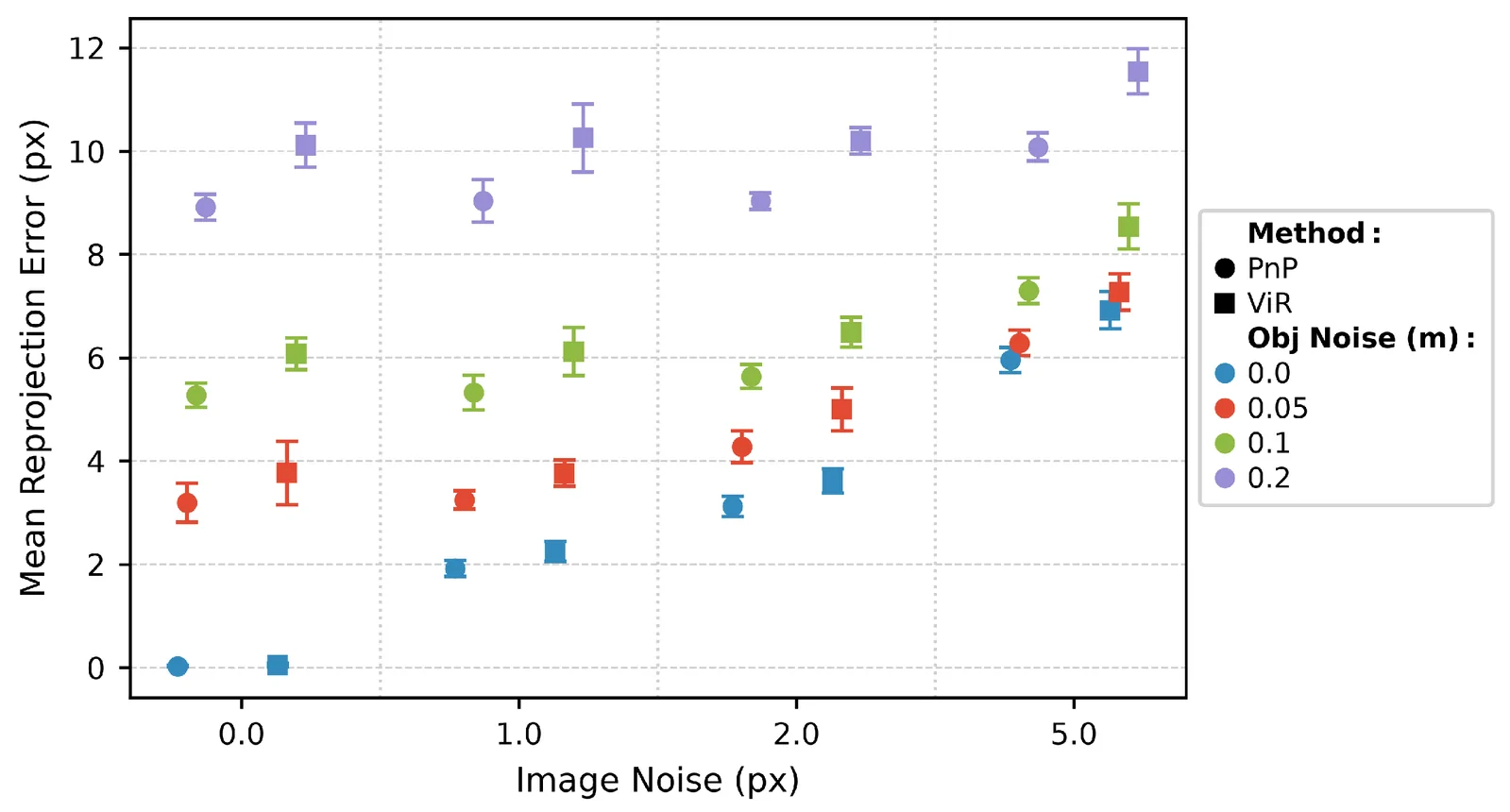
Reprojection error with 95% confidence interval.

In Figure 14, the mean reprojection error is substantially lower than the real-world results. Image noise and object noise consistently increases mean reprojection error. Additionally, these synthetic results are consistent with the real-world results as the planar PnP algorithm performs slightly better than our ViR algorithm regardless of noise. Overall, mean reprojection error is consistently below 12 pixels.

#### 3.2.3. Synthetic: Rotation Estimation and Relative Translation Error

**Figure 15:**
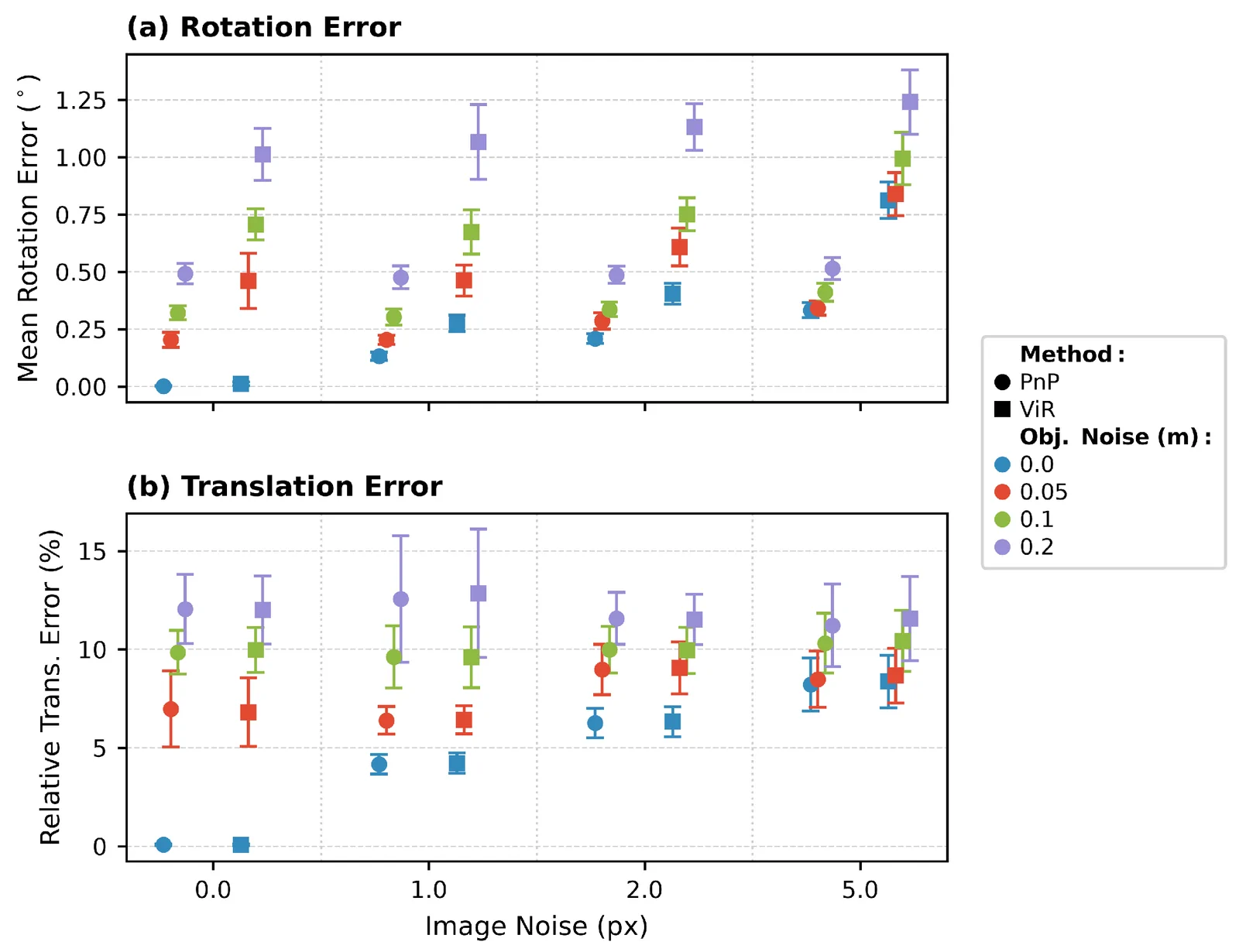
(**a**) Rotational error and (**b**) relative translation error with 95% confidence interval.

The rotation estimation error (Figure 15a) appears to be below $1^{{}^{\circ}}$ regardless of noise or extrinsic camera parameter estimation methodology. Image and object noise both contribute to rotation estimation error. The planar PnP once again is more accurate and robust to noise compared with our ViR method.

The relative translation error is consistently below 15% (Figure 15b). The trend of image noise and object noise is consistent with the error in focal length estimation; high levels of image noise produces a lower floor for translation estimation accuracy. Overall, the relative translation error in the synthetic data reflects the relative translation error of the longer trajectories in the real-world data.

## 4. Discussion

In this section, we discuss possible explanations for particular results and provide further analysis of the methodology, possible solutions for improving performance of the algorithms, and potential future work.

### 4.1. Camera Pose Error

In Figure 9, the results indicate that the reprojection error is lower in shorter trajectories with fewer points to sample from, while the rotation (Figure 10) and translation (Figure 11) indicate that shorter trajectories result in greater camera pose error. An explanation for this result is that utilizing the minimum reprojection error as a selection mechanism for extrinsic camera parameters does not necessarily result in a lower estimation error, as it is merely a proxy for both intrinsic and extrinsic camera parameters. In Table 4, we can see that even from the best estimate by the extrinsic parameter estimator, the mean reprojection error does not necessarily correlate with better camera pose estimation.

**Figure 16:**
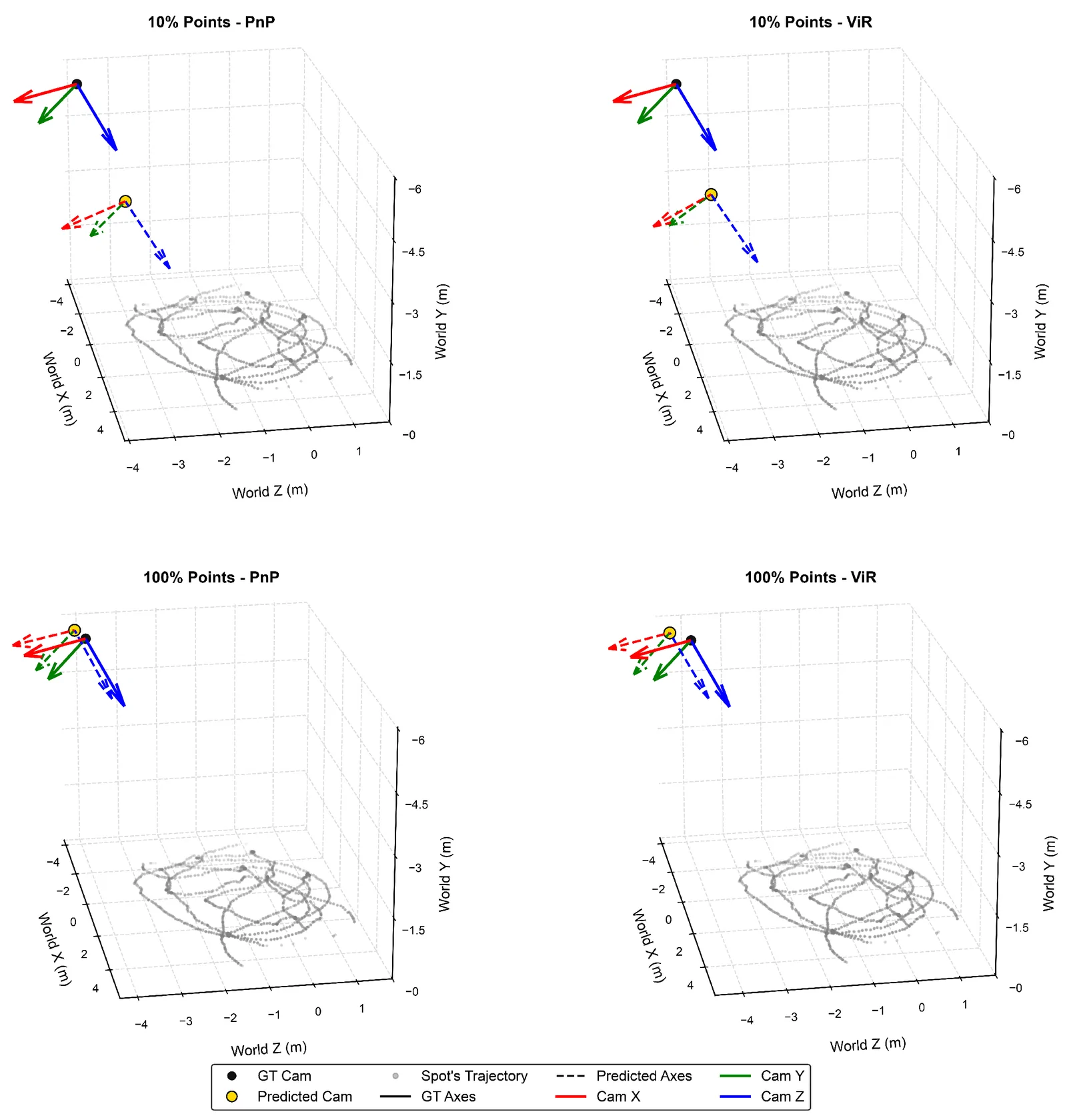
Camera pose by percentage of points utilized and extrinsic parameter estimation methodology compared to ground-truth camera pose.

Figure 16 illustrates the best estimated camera pose of both extrinsic camera parameter estimation algorithms utilizing only the first 10% of Spot’s trajectory in the top row where both estimates are far from the ground-truth camera pose. In the bottom row, both estimation algorithms utilizing Spot’s full trajectory produce reasonably close estimates to the ground-truth.

In Figure 11, we have previously observed that the relative translation error appears to decrease when we utilize the ground-truth focal length for extrinsic camera parameter estimation. This suggests that increasing the accuracy of the focal length estimation should increase the accuracy of camera translation estimation in a global coordinate frame as scale is affected by focal length.

### 4.2. Synthetic Results in Explaining Real-World Results

The synthetic results provide a reference point for finding the real-world conditions that may contribute to estimation errors. In Section 2.1, from prior calibration, the self-localization error of Spot is less than 22 cm based on the length of the trajectory. In Figure 11, we have previously observed in the real-world results that relative translation error is trending towards 10% (and below in the case of a very accurate focal length estimation) while in Figure 15b, this aligns with image noise of 2 pixels and object noise between 5 and 10 cm. Evaluating the keypoint tracking of Spot (Figure 5b) also aligns with these results of roughly 2 to 5 pixels of image noise.

### 4.3. Potential Improvements

**Figure 17:**
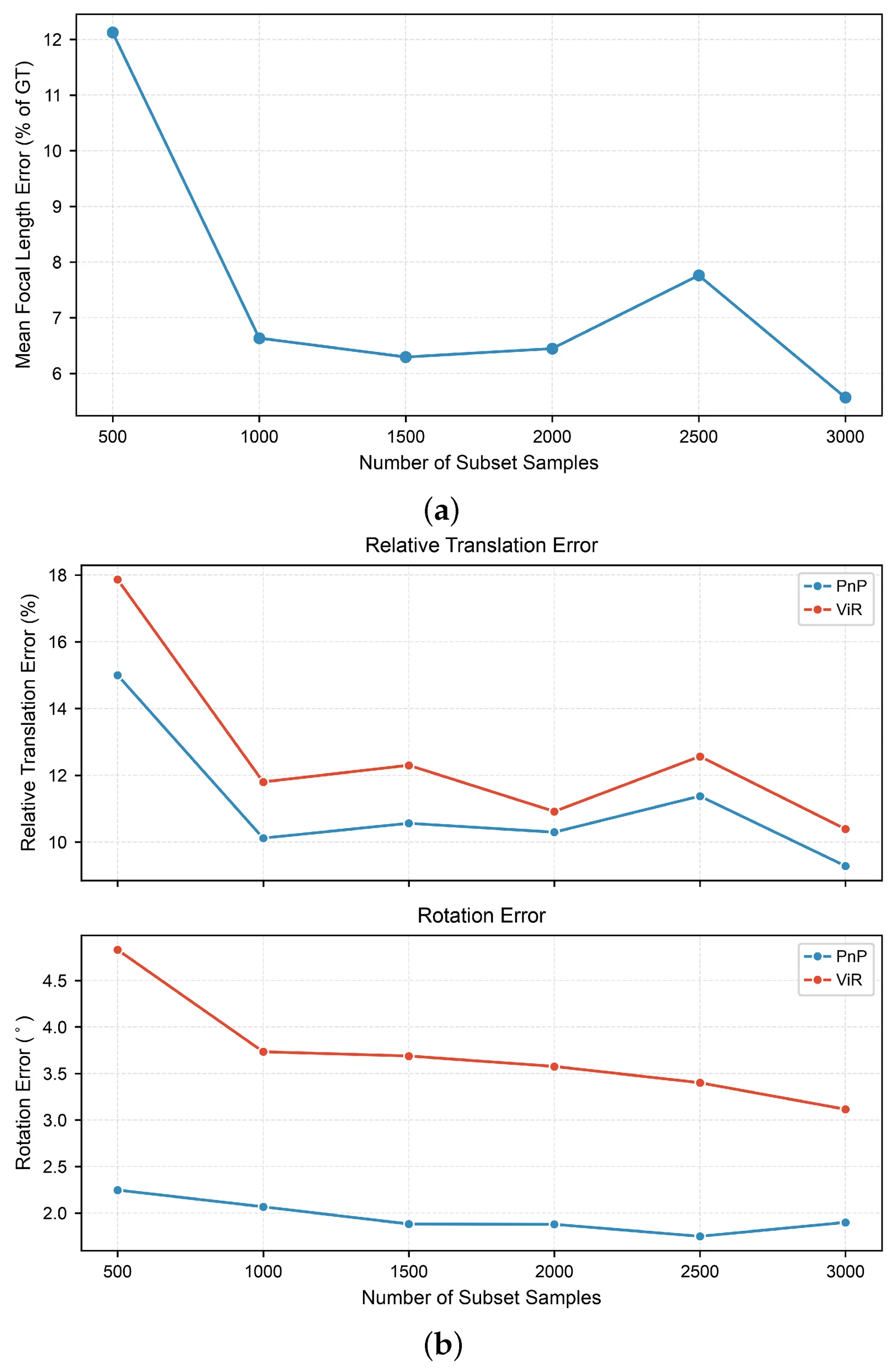
(**a**) Focal length estimation error vs. number of subset samples; (**b**) translation and rotation error vs. number of subset samples.

Due to computational time, we selected 500 subsets of points to test our algorithm across 100 independent trials for the real-world and synthetic data. We provide a more detailed analysis in terms of number of subset samples. In Figure 17a, we observe (from real-world data) that the number of subsets sampled correlates with better focal length estimation. A total of 3000 subsets produces a mean focal length error across 50 independent trials below 75 pixels. This suggests that a larger number of sampled subsets may lead to a more accurate focal length estimation. It is also shown (Figure 17b) that increasing the number of sampled subsets generally improves camera pose error.

One drawback of increasing the number of sampled subsets is increased computational time, this increase scales linearly. Both the focal length estimation stage and the extrinsic camera parameter estimation stage have a linear runtime relation to the number of subsets.

### 4.4. Future Work

The first step in future work is to begin testing the efficacy of our system in a real-time indoor environment as shown in Figure 1. This can be accomplished by identifying static objects of known heights and instructing Spot to the object’s estimated position within Spot’s coordinate system (SLAM/LiDAR) utilizing this paper’s algorithm.

For potential future work on improving object point acquisition from the trajectory, as compared to the DLO method for the 3D ground-truth pose acquisition in this study, we acknowledge that several later developed LiDAR-based SLAM algorithms may perform with higher accuracy. For instance, the DLIO method almost doubled the accuracy in pose estimation [26]. The reason the DLO method was employed by our study is primarily due to the hardware constraints as our robot-carried Velodyne VLP-16 sensor generates sparse point cloud frames, which may significantly degrade the pose estimation if using other LiDAR-based SLAM approaches (e.g., DLIO). Future work includes evaluating the hardware compatibility and applicability between other potentially more accurate SLAM methods and our application for further improvements in 3D pose data acquisition.

We will also be testing longer trajectories for Spot. From the observation of the results, there appears to be room for improvement with a longer trajectory. Allowing Spot to traverse distances greater than the current trajectory of approximately 100 m may allow a more detailed analysis of the optimal trajectory distance. Additionally, we would like to run further studies utilizing the estimated camera calibration to determine the localization accuracy of the BLV individual in a large public indoor setting.

## 5. Conclusions

In this paper, we present a novel approach for dynamically calibrating the focal length and extrinsic parameters of static surveillance cameras by utilizing a quadruped robot as a mobile calibration agent. This is especially useful since the approach only needs the robot walking on a planar surface, which is usually the case in real-world applications, such as in a stadium or a museum. By extracting virtual geometric structures from the robot’s dynamic planar trajectory, our method successfully bridges the gap between robotic 3D spatial data and 2D camera feeds without manual calibration targets. While our full end-to-end planar methodology offers a unified calibration approach, it is slightly weaker than the standard planar Perspective-n-Point (PnP) algorithm, yielding marginally higher reprojection, rotational, and translational errors. However, because its performance remains highly comparable, the full planar algorithm serves as an effective, standalone alternative for end-to-end calibration in software frameworks constrained by library dependencies.

Crucially, the most significant contribution of our approach lies in its first phase: the focal length estimation. Because highly efficient algorithms like PnP are generally extrinsic-only solvers that require known focal length to function accurately, our focal length estimation step acts as a robust, drop-in prerequisite module. By accurately recovering the focal length, this step allows the use of standard PnP solvers on entirely uncalibrated, heterogeneous camera networks. Ultimately, repurposing existing surveillance infrastructure provides a scalable pathway to enhance real-time spatial accessibility for blind and low-vision individuals in public facilities, with future work investigating the impact of significantly longer robotic trajectories (exceeding 200 m) to further refine estimation accuracy.

## Figures and Tables

**Table 1:** Camera pose ground-truth parameter baseline.

Component	Translation (m)			Rotation (°)		
	X	Y	Z	Yaw	Pitch	Roll
Camera	−3.89	−5.48	−3.83	55.5	−45.8	0.0

**Table 2:** First *X*% of trajectory with corresponding distance and number of points on trajectory.

% of Trajectory	10	20	30	40	50	60	70	80	90	100
Meters	10.1	16.9	24.0	33.1	41.1	49.6	57.6	66.8	74.0	81.1
Points	307	614	921	1227	1534	1841	2147	2454	2761	3067

**Table 3:** Virtual camera pose ground-truth parameters.

Component	Translation (m)			Rotation (°)		
	X	Y	Z	Yaw	Pitch	Roll
Camera	−3	−11	−20	10	−30	10

**Table 4:** Best estimates by methodology.

Estimator	Proportion of Points	Mean Reprojection Error (px)	Translation Error (m)	Rotational Error (°)
PnP	10%	10.99	3.43	6.09
	100%	21.56	0.44	1.46
ViR	10%	17.28	3.45	10.76
	100%	21.93	0.56	1.50

## Data Availability

The original contributions presented in this study are included in the article. Further inquiries can be directed to the corresponding author.

## Abbreviations

The following abbreviations are used in this manuscript:

BLV	Blind or Low Vision
ViR	Virtual Rectangle
PnP	Perspective-n-Point
LLM	Large Language Model
IMU	Inertial Measuring Unit
RANSAC	Random Sample Consensus
VP	Vanishing Point
FOV	Field of View
3D	Three-Dimensional
2D	Two-Dimensional
